# Portal venous circulating tumor cells as a biomarker for relapse prediction in resected pancreatic cancer

**DOI:** 10.1007/s00018-025-05669-x

**Published:** 2025-04-10

**Authors:** Dannel Yeo, Doruk Seyfi, Althea Bastian, Heidi Strauss, Anna Leach, Vera Klemm, Anthony Pirrello, Kevin Spring, Payal Saxena, Sara Wahlroos, Sarah Sutherland, Peter Grimison, Jin-soo Park, Charbel Sandroussi, John EJ Rasko

**Affiliations:** 1 Li Ka Shing Cell & Gene Therapy Program, The University of Sydney, Camperdown, Australia; 2 Precision Oncology Laboratory, Cancer Innovations, Centenary Institute, Camperdown, Australia; 3 Faculty of Medicine and Health, The University of Sydney, Camperdown, Australia; 4 Cell and Molecular Therapies, Royal Prince Alfred Hospital, Sydney Local Health District, Camperdown, Australia; 5 Department of Hepatobiliary and Upper Gastrointestinal Surgery, Royal Prince Alfred Hospital, Sydney Local Health District, Camperdown, Australia; 6 Upper Gastrointestinal Surgery, Chris O’Brien Lifehouse, Camperdown, Australia; 7 Medical Oncology Group, Liverpool Clinical School, School of Medicine, Western Sydney University and Ingham Institute for Applied Medical Research, Liverpool, Australia; 8 Concord Institute of Academic Surgery, Sydney Local Health District, Concord, Australia; 9 South-West Sydney Clinical Campuses, UNSW Medicine & Health, Sydney, Australia; 10 Division of Gastroenterology, Department of Medicine, Royal Prince Alfred Hospital, Sydney Local Health District, Camperdown, Australia; 11 Gastroenterology, Chris O’Brien Lifehouse, Camperdown, Australia; 12 Medical Oncology, Chris O’Brien Lifehouse, Camperdown, Australia

**Keywords:** Liquid biopsy, Biomarkers, Risk assessment, Precision medicine, Portal venous, Minimal residual disease

## Abstract

**Background:**

Pancreatic cancer is an aggressive disease with poor prognosis. The only potentially curative treatment option is surgical resection, however recurrence is common. Biomarkers to detect minimal residual disease, assist with risk stratification, relapse and real time monitoring, are required. Circulating tumor cells (CTCs) are a promising liquid biopsy biomarker for solid tumors. However, their role in monitoring minimal residual disease in pancreatic cancer remains to be determined. Our study aimed to investigate whether detection and enumeration of CTCs could predict recurrence and provide monitoring of disease status.

**Method:**

Participants planned for Whipple procedure or partial pancreatectomy were enrolled in this prospective pilot study. Intraoperatively, 7.5 mL of portal and peripheral venous blood were collected, and peripheral venous blood was also collected post-surgery. CTC identification and enumeration were performed using the AccuCyte-CyteFinder platform and CellSieve microfiltration.

**Results:**

Of 29 participants, 20 were confirmed to have epithelial cancer by histopathology, where 15 had pancreatic ductal adenocarcinoma. In those with epithelial cancer, CTCs were detected intraoperatively in 75% of portal venous blood samples, in contrast to 40% detected in peripheral venous blood (median: 6 and 0 per 7.5mL respectively). Only portal venous CTC detection was predictive of pancreatic ductal adenocarcinoma relapse. The positive (> 5) portal venous CTC group had a 6.67 times higher risk of recurring (odds ratio = 20.43, sensitivity = 1.00, specificity = 0.625). Detection of peripheral venous CTCs post-surgery was also correlated with relapse in a small subset of patients.

**Conclusions:**

If validated, CTCs may provide a prognostic and monitoring biomarker in patients with pancreatic cancer undergoing surgery.

**Supplementary Information:**

The online version contains supplementary material available at 10.1007/s00018-025-05669-x.

## Background

Pancreatic ductal adenocarcinoma (PDAC) is an aggressive disease with poor prognosis. It accounts for approximately 8.5% of all cancer-related deaths with an overall 5-year survival rate of ~ 12% [1]. Incidence and mortality rates are expected to increase, leading to it becoming the second leading cause of cancer-related death by 2040 [2, 3]. Surgical resection remains the only potentially curative treatment option for PDAC patients. However, only 13.6% of patients present with localized disease [4]. For those that do present with resectable disease, recurrence rates remain high with up to 40% of patients recurring within 1 year from surgery [5]. The overall survival (OS) for localized disease after surgery has improved with the incorporation of adjuvant FOLFIRINOX chemotherapy (combination of folinic acid, fluorouracil (5-FU), irinotecan, and oxaliplatin) [6]. Biomarkers to predict and detect early recurrence for optimal patient management are needed.

The blood biomarker serum carbohydrate 19 − 9 antigen (CA19-9) is a widely used tumor marker for PDAC [7]. CA19-9 levels correlate with tumor size, stage and burden of disease [8], and pre- and postoperative CA19-9 levels may predict prognosis [9]. Monitoring the dynamic changes in CA19-9 levels is clinically informative during chemotherapy [10] and for surveillance post-surgery [11]. However, there are several limitations such as a subpopulation (~ 10%) that does not express CA19-9 and the elevation of CA19-9 in those with benign diseases [12, 13]. Hence, PDAC recurrence is mostly diagnosed using imaging techniques such as computed tomography (CT) scan, endoscopic ultrasound, or magnetic resonance imaging (MRI) [14].

Circulating tumor cells (CTCs) are malignant cells present in the bloodstream, originating from the tumor. They are understood to be the ‘seeds’ of metastases where cells shed by the primary or secondary tumor circulate through the blood, and colonize distant sites [15]. The prognostic value of CTCs has been demonstrated in various cancer types including PDAC [16–19]. There is currently no gold standard CTC detection method. CellSearch is a commonly used FDA-approved method that involves immunomagnetic separation using the epithelial marker EpCAM followed by confirmatory cytokeratin (CK) staining [20]. In contrast, the high-resolution image scanning platform AccuCyte CyteFinder involves spreading enriched nucleated blood cells on a slide, followed by staining and identification using image analysis based on marker(s) expression [21]. AccuCyte-CyteFinder has been shown to be equivalent to CellSearch in detecting CTCs in metastatic breast cancer [22]. The microfiltration device, CellSieve isolates cells based on size exclusion. CellSieve identifies distinct CTC subpopulations that are not identified by CellSearch in addition to being more likely to detect CTC clusters [23, 24] and cancer-associated macrophage-like cells [25].

CTCs are generally detected from peripheral venous blood samples due to the ease of access to peripheral veins. However, peripheral venous sampling may limit CTC detection. In PDAC, venous drainage occurs first through the portal circulation. Hence, portal venous blood may be more suitable for detecting PDAC CTCs [26]. The detection rate and number of CTCs have been found to be higher in portal venous blood compared to peripheral venous blood. Portal venous CTC counts have been associated with hepatic metastasis, recurrence after surgery, and survival [27–29]. However, several studies did not demonstrate a difference in the CTC detection rates between portal and peripheral venous blood samples [30, 31]. No studies, to our knowledge, have used the AccuCyte-CyteFinder platform to detect CTCs from portal venous blood samples.

The aim of this prospective observational pilot study was to evaluate the clinical utility of CTCs to identify recurrence, by correlating the detection of CTCs intraoperatively and post-operatively with clinicopathological factors and clinical outcomes. The performance of detecting CTCs using AccuCyte and CellSieve microfiltration was also compared.

## Methods

### Study participants

This prospective observational pilot study recruited participants who were planned for Whipple procedure (pancreatoduodenectomy) or partial pancreatectomy following confirmation of pancreaticobiliary malignancy. This study was conducted in accordance with the Declaration of Helsinki and was approved by the Sydney Local Health District Ethics Committee (X19-0490). All subjects provided written informed consent prior to participation. A total of 29 participants were identified and enrolled between July 2020 and February 2024 at Royal Prince Alfred Hospital and Chris O’Brien Lifehouse, Australia.

### Clinical data and sample collection

Data were collected for age, gender, site of primary tumor, tumor size, CA19-9 level, clinical treatment and outcomes. TNM staging was performed according to The American Joint Committee on Cancer (AJCC) 8th Edition 2017. Follow up clinical data was collected up to 31st May 2024.

Blood samples were collected in AccuCyte^®^ blood collection tubes (RareCyte, Seattle, WA, USA). Baseline peripheral venous blood was collected at the time of anaesthesia. Baseline portal venous blood was collected intraoperatively by direct puncture via a 21-gauge needle before manipulation of the tumor. Post-surgery follow-up blood samples were collected at least 7 days prior to any infusion of therapy. Blood samples were processed between 24 and 72 hours after collection.

### CTC isolation and enumeration using AccuCyte-CyteFinder platform

Blood samples (7.5 mL) were processed using the AccuCyte-CyteFinder platform, as previously described [21]. Briefly, blood nucleated cells were isolated using the AccuCyte system (RareCyte) and spread onto 8 Superfrost^®^ Plus slides (Thermo Scientific) using a plastic CyteSpreader^®^ device (RareCyte). Two slides (representing approximately 6 million nucleated cells) were immunofluorescently stained on a Autostainer Link 48 (Dako–Agilent Technologies) using the RarePlex 0700-MA staining kit (RareCyte) comprising a nuclear DAPI dye, anti-pan-cytokeratin (CK) antibody conjugated to CF^®^488, anti-EpCAM antibody conjugated to CF^®^647, and anti-CD45 antibody conjugated to R-phycoerythrin (PE). Stained slides were scanned on the CyteFinder digital immunofluorescent microscope (10X magnification; RareCyte), at exposure times of 0.05s (DAPI), 0.025s (CK), 0.1s (EpCAM), and 0.1s (CD45). Image files were analyzed using the CyteMapper^®^ software (RareCyte) and then reviewed by trained users (AB, HS; and confirmation by DY). A CTC was defined as having a nucleus, CK and/or EpCAM staining (mean fluorescence intensity (MFI) cutoff of 15 for determining high/low expression) and no CD45 staining. CTC counts were normalised to 7.5mL blood for comparisons as per routine usage.

### CTC isolation and enumeration using the cellsieve microfiltration assay

Blood samples were also processed on the CellSieve Microfiltration Assay using a low-pressure vacuum system, as previously described [32]. Briefly, 7.5 mL of blood was fixed in a pre-fixation buffer (Creatv MicroTech) and then trasnferred into a 30 mL syringe. Samples were filtered through a medical pump (Chemyx Incorporated) containing a pre-prepared membrane, consisting of a uniform array of 160,000 7 μm pores in a 9 mm diameter area, at 2 mL/ min (Creatv MicroTech). The membrane was stained using a nuclear DAPI dye (Invitrogen), anti-pan-cytokeratin (CK) antibody conjugated to Alexa Fluor-488 (MA5-18156, Invitrogen), anti-EpCAM antibody conjugated to Alexa Fluor-488 (324210, BioLegend), and anti-CD45 antibody conjugated to PE (304008, BioLegend). The membrane was mounted onto a Superfrost^®^ Plus slide (Thermo Scientific) using an aqueous mounting media (RareCyte) and scanned on the CyteFinder digital immunofluorescent microscope (10X magnification; RareCyte), at exposure times of 0.05s (DAPI), 0.05s (CK/EpCAM), and 0.1s (CD45). Image files were analyzed using the CyteMapper^®^ software (RareCyte) and then reviewed by trained users as above. A CTC was defined as having a nucleus, CK or EpCAM staining and no CD45 staining.

### Generating spike-in samples

AsPC-1, a pancreatic cancer cell line, was obtained from American Type Culture Collection (ATCC, Manassas, VA, USA) and maintained in RPMI 1650 medium supplemented with 10% FBS. Cells were cultured in a fully humidified incubator maintained at 37 °C with 5% CO2. Human donor blood samples were obtained from healthy volunteers after informed consent (Sydney Local Health District, Australia: X19-0321). Surrogate CTC samples were generated using the Indirect-Supplement method, as previously described [21]. Briefly, the volume of spike-in cells was calculated to achieve the target cell number and added directly to the blood collection tube. Samples were then processed on the Accucyte-CyteFinder platform or CellSieve Microfiltration assay as described above.

### Data analysis

Statistical analysis was undertaken using GraphPad Prism version 10.0.3. Paired student’s t test (2 tailed) was used to determine p values for comparison of CTC numbers identified from portal and peripheral venous blood samples or between the two CTC detection methods. Unpaired student’s t test (2 tailed) was used to determine p values when comparing CTC numbers and patient clinical characteristics. Specificity was calculated by total number of true negatives (non-epithelial cancer and other malignancy group with no CTCs) divided by the total number of individuals in the group (non-epithelial cancer and other malignancy group). A cutoff of greater than 5 CTCs was used to define CTC-positivity. Contingency analysis for two variables was undertaken using Fisher’s exact test with the Woolf method for odd’s ratio analysis and the Katz method for relative risk analysis. The chi-square test was performed when testing for significance with three or more variables.

## Results

### Patient characteristics

**Fig. 1:**
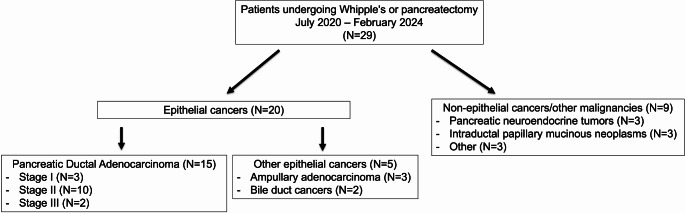
Study subgroups based on the pathological diagnosis

69% (20/29) of the study population comprised individuals with epithelial cancers and 31% (9/29) with non-epithelial cancers/other malignancies (Table 1; Supp Table 1). Of the participants with epithelial cancers, 15 were PDAC (3 stage I, 10 stage II, and 2 stage III), 3 ampullary adenocarcinomas (AA) and 2 bile duct (BD) cancers (Fig. 1). Of the non-epithelial cancers/other malignancies, 3 were pancreatic neuroendocrine tumors (pNET), 3 intraductal papillary mucinous neoplasms (IPMN), and 3 others (1 pseudopapillary tumor, 1 serous cystadenoma, and 1 gastrointestinal stromal tumor (GIST)). Elevated CA19-9 levels (> 37 U/mL) were present pre-operatively in 55% (11/20) of the epithelial cancer group but none in the non-epithelial/other malignancies group. In the epithelial cancer group, 2 participants received neoadjuvant chemotherapy: PaCa5 received FOLIRINOX with stereotactic body radiation therapy (SBRT) and PaCa12 received modified FOLIRINOX; and 12 patients received adjuvant chemotherapy. No patients in the non-epithelial cancer/other malignancies group received neoadjuvant or adjuvant therapy.

**Table 1:** Participant and tumor characteristics

Parameters	Epithelial cancer group (*N* = 20)	Non-epithelial cancer/other malignancies group (*N* = 9)
Age mean (range)	70 (42–85)	61 (48–80)
Gender, male (%)	16 (80)	5 (56)
CA19-9, median (IQR) U/mL	77 (13.25–468)	9 (8.5–12.5)
Pancreaticoduodenectomy (%)	18 (90)	9 (100)
R0 residual tumor classification (%)	15 (75)	9 (100)
Vascular reconstruction (%)	6 (30)	0 (0)
Neoadjuvant therapy (%)	2 (10)	0 (0)
Adjuvant therapy (%)	12 (60)	0 (0)
Tumor stage		
Stage I (%)	5 (25)	1 (11)
Stage II (%)	10 (50)	3 (33)
Stage III (%)	5 (25)	0 (0)
Stage IV (%)	0 (0)	0 (0)

### CTC detection in portal and peripheral venous blood samples

**Fig. 2:**
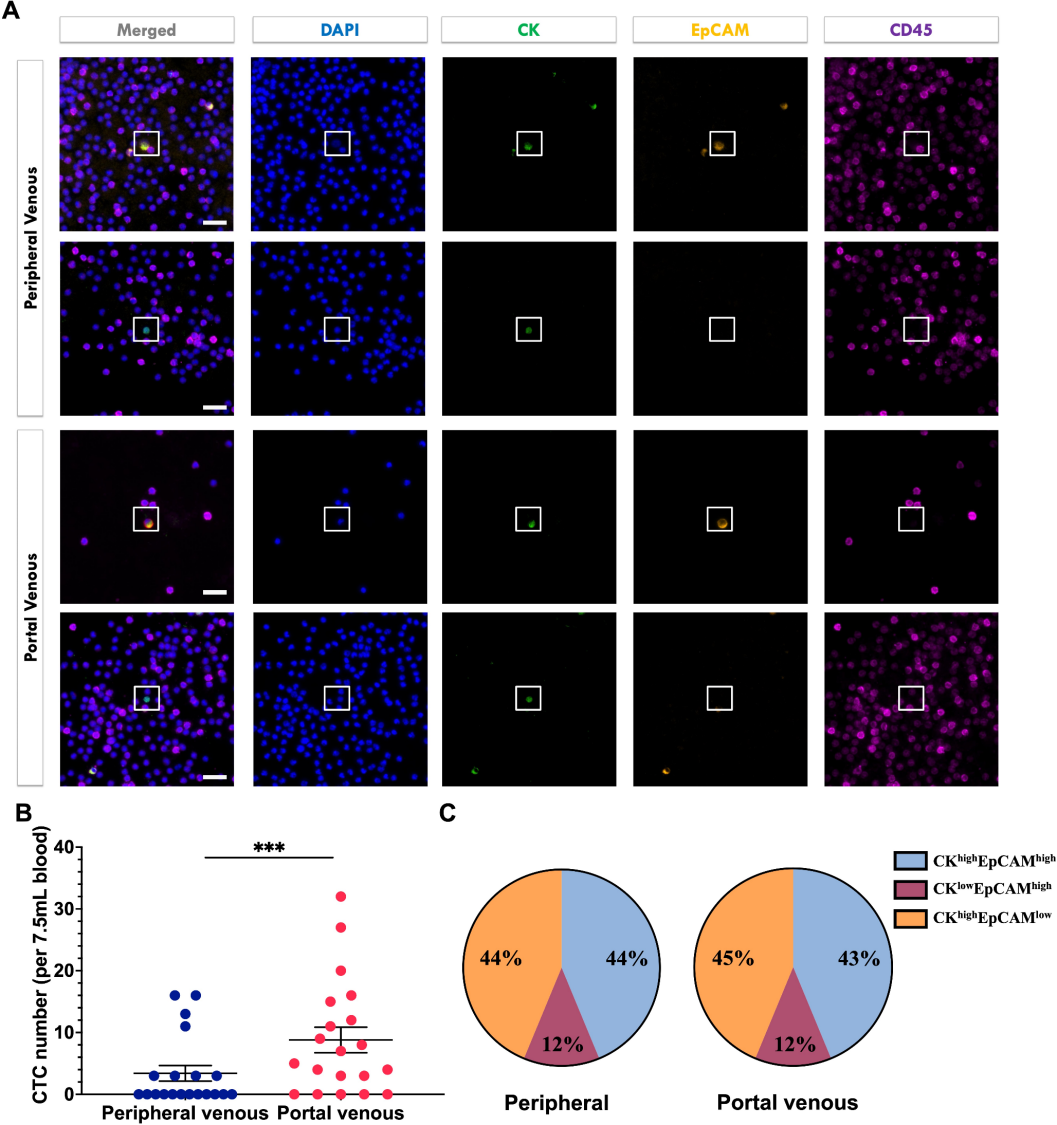
Representative images of circulating tumor cells (CTCs) from peripheral and portal venous blood samples expressing cytokeratin (CK) and/or EpCAM (**A**). Magnification 40x, scale bar = 50 μm. CTC mean counts in participants with epithelial cancers were significantly higher in the intra-operative portal venous blood samples compared to intra-operative peripheral venous blood samples (**B**). Line indicates mean ± SEM. *** p<0.001. Similar proportions of CTCs expressing CK and/or EpCAM were identified in peripheral and portal venous blood samples (**C**)

In the epithelial cancer group (*N* = 20), CTCs were detected in 75% (15/20) of baseline portal venous blood samples, and 40% (8/20) of baseline peripheral venous blood samples (​Table 2). Mean CTCs counts in portal venous blood were significantly higher than those in peripheral venous blood (8.8 vs. 3.4 per 7.5 ml, *p* = 0.005, Figure ​2; Supp Table 2). Similar proportions of CTC expressing CK and EpCAM were found in portal and peripheral venous blood samples: CK^high^EpCAM^high^ (43% vs. 44%), CK^high^EpCAM^low^ (45% vs. 44%), and CK^low^EpCAM^high^ (12% vs. 12%) (Fig. 2C; Supp Table 3).

**Table 2:** CTC counts in intra-operative peripheral and portal venous blood samples

Patient ID	Cancer	Stage	Pre-operative CA19-9 (U/mL)	CTCs (in 7.5 mL blood)	
				Peripheral Venous	Portal Venous
**Epithelial Cancer Group**					
PaCa1	PDAC	1	9	0	0
PaCa2	PDAC	1	9	0	4
PaCa3	PDAC	1	458	0	0
PaCa4	PDAC	2	3	0	3
PaCa5	PDAC	2	14	0	0
PaCa6	PDAC	2	21	0	3
PaCa7	PDAC	2	24	11	11
PaCa8	PDAC	2	131	0	8
PaCa9	PDAC	2	202	0	0
PaCa10	PDAC	2	574	0	0
PaCa11	PDAC	2	636	3	7
PaCa12	PDAC	2	779	0	5
PaCa13	PDAC	2	1810	16	16
PaCa14	PDAC	3	96	0	20
PaCa15	PDAC	3	498	13	15
PaCa16	AA	1	8	3	4
PaCa17	AA	1	11	0	12
PaCa18	AA	3	23	3	32
PaCa19	BD	3	58	16	27
PaCa20	BD	3	226	3	9
**Non-epithelial Cancer/Other Malignancies Group**					
PaCa21	IPMN	NA	5	0	5
PaCa22	IPMN	NA	12	0	0
PaCa23	IPMN	NA	20	0	0
PaCa24	pNET	1	9	0	0
PaCa25	pNET	2	-	0	0
PaCa26	pNET	2	13	0	0
PaCa27	Other^a^	NA	8	0	0
PaCa28	Other^b^	NA	9	0	0
PaCa29	Other^c^	NA	-	0	0

CTC: circulating tumor cell; PDAC: pancreatic ductal adenocarcinoma; AA: ampullary adenocarcinoma; BD: bile duct cancer; IPMN: intraductal papillary mucinous neoplasm; pNET: pancreatic neuroendocrine tumor; ^a^: gastrointestinal stromal tumor; ^b^: pseudopapillary tumor; ^c^: serous cystadenoma

**Table 3:** Correlation between clinical parameters and CTC counts in PDAC patients

Characteristics	Peripheral venous CTC counts (mean, 95%CI)	*p* value	Portal venous CTC counts (mean, 95%CI)	*p* value
Age (years)				
≤70 >70	1.57 (-2.28, 5.42) 4 (-1.53, 9.53)	ns	6.13 (-0.47, 12.76) 6.14 (0.63, 11.62)	ns
Gender				
Male Female	3.00 (-1.68, 7.68) 2.67 (-2.79, 8.13)	ns	6.78 (1.04, 12.51) 5.17 (-0.67, 11.01)	ns
CA19-9 levels				
<37 >37	1.83 (-2.88, 6.55) 4.00 (-1.53, 9.53)	ns	3.50 (-0.74, 7.74) 6.38 (0.96, 11.79)	ns
Stage				
1 2 3	0.00 (0.00, 0.00) 3.00 (-1.10, 7.10) 6.50 (-76.09, 89.09)	ns	1.33 (-4.40, 7.07) 5.30 (1.51, 9.09) 17.50 (-14.27, 49.27)	0.009
Tumor size (mm)				
≤30 >30	1.78 (-2.32, 5.88) 5.40 (-2.28, 13.08)	ns	3.44 (-0.75, 7.64) 8.20 (2.22, 14.18)	ns
Lymphovascular invasion				
No Yes	0.00 (0.00, 0.00) 3.58 (-0.25, 7.42)	ns	4.50 (-1.85, 10.85) 5.25 (1.41, 9.09)	ns

CTC: circulating tumor cell; ns: not significant

In the non-epithelial cancers/other malignancies group (*N* = 9), CTCs were only detected in the portal venous blood sample of one patient (PaCa21 with main duct IPMN), resulting in a specificity of 89%. No CTCs were detected in any of the peripheral venous blood samples (Table 2).

### Clinical correlations with CTCs in PDAC patients

In PDAC patients (*N* = 15), comprehensive clinical correlations with CTC counts were examined (Table 3). Portal venous CTC counts were correlated with tumor stage (*p* = 0.009) (Supp Fig. 1) but not CA19-9 level or other clinical parameters. Peripheral venous CTC counts were not significantly correlated with the clinical parameters.

### Prognostic value of portal venous CTCs in PDAC patients

**Fig. 3:**
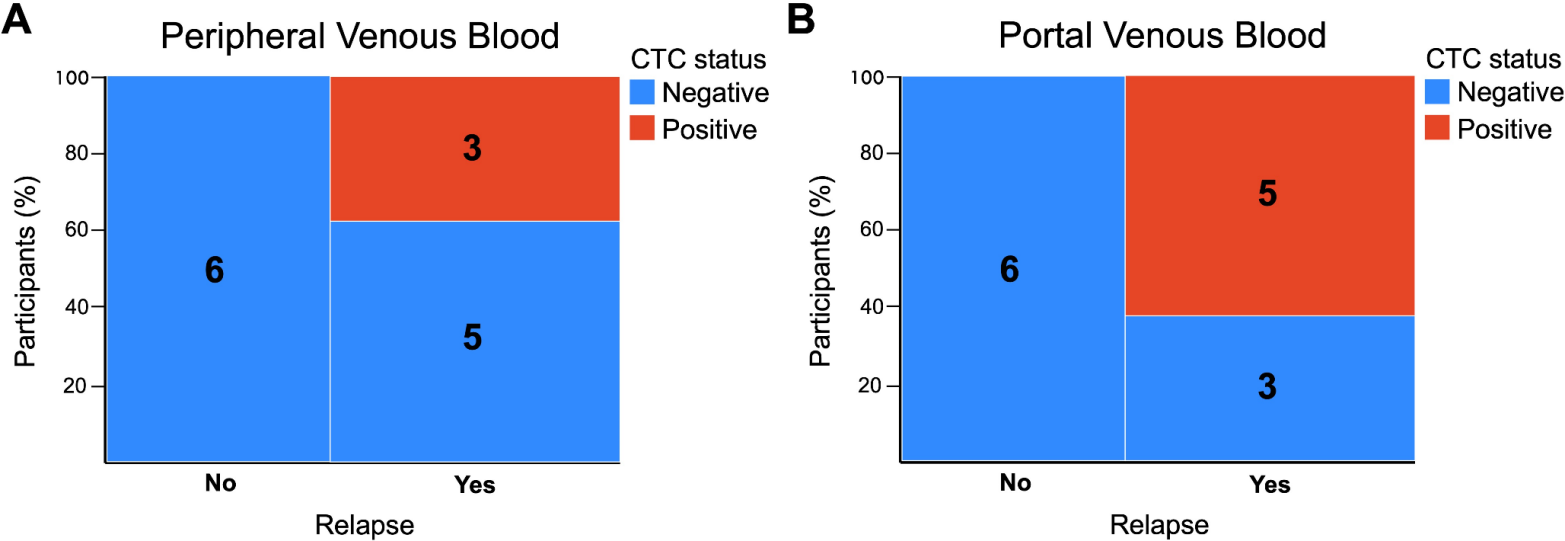
Contingency table of baseline circulating tumour cell (CTC) status (positivity defined by > 5 CTCs per 7.5mL) from peripheral venous blood (**A**) and portal venous blood (**B**) in participants who subsequently relapsed. Number of patients in each category are listed

In the PDAC patient group where follow-up clinical data was available (*N* = 14), 57% (8/14) of participants relapsed during the 12-months post-surgery. A cutoff of greater than 5 CTCs was used to define CTC-positivity. All participants (100%; 5/5) with a positive baseline portal venous CTC sample relapsed compared to 33% (3/9) of those who were negative (odds ratio = 20.43, relative risk = 6.67, *p* = 0.031) (Fig. 3). All participants (100%; 3/3) with a positive baseline peripheral venous CTC sample relapsed compared to 45% (5/11) of those who were negative (odds ratio = 8.27, relative risk = 3.27, *p* = 0.2). None of the other clinicopathological factors predicted relapse (Table 4). Recurrence-free survival was not significantly different in subjects either with CTC positive portal venous or CTC positive peripheral venous samples (Supp Fig. 2).

**Table 4:** Contingency analysis of clinicopathological factors to predict relapse in PDAC patients

Characteristics	Disease-free (*N* = 6)	Relapse (*N* = 8)	*p* value
Age (years)			
≤70 >70	3 3	3 5	ns
Gender			
Male Female	4 2	4 4	ns
CA19-9 level (U/mL)			
≤37 >37	3 3	3 5	ns
Tumor stage			
1 2 3	3 3 0	0 7 1	ns
Tumor size (mm)			
≤30 >30	5 1	4 4	ns
Lymphovascular invasion			
No Yes	2 4	0 8	ns
Vascular reconstruction			
No Yes	5 1	3 5	ns
Residual tumor classification			
R0 R1	4 2	6 2	ns
Peripheral venous CTCs			
≤5 >5	6 0	5 3	ns
Portal venous CTCs			
≤5 >5	6 0	3 5	0.031

### Longitudinal monitoring of CTCs in PDAC

**Fig. 4:**
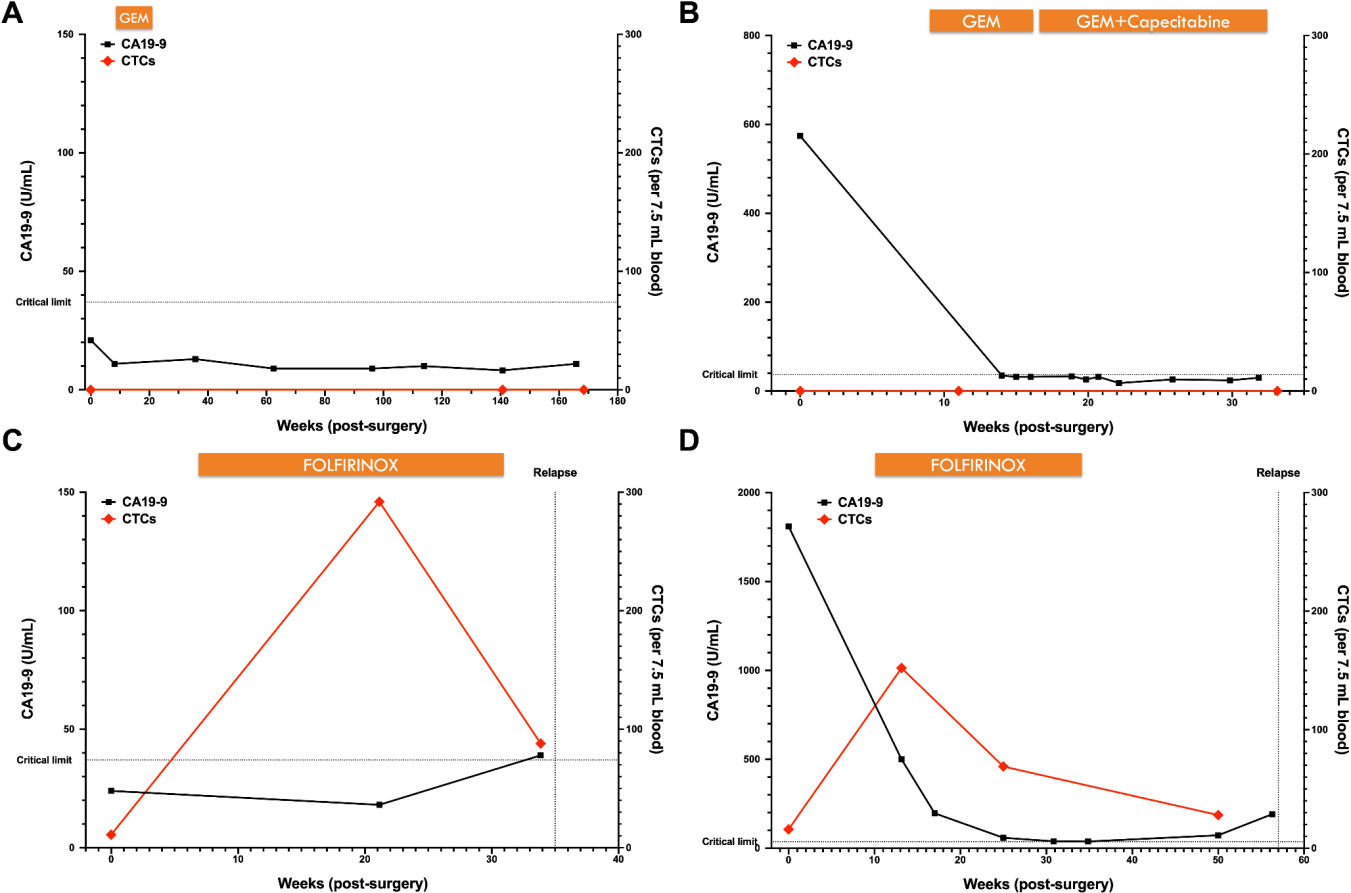
Peripheral venous blood biomarkers: CA19-9 (left y-axis) and circulating tumor cell (CTC; right y-axis) enumeration post-surgery from participants with pancreatic ductal adenocarcinoma. Both participants, PaCa6 (**A**) and PaCa13 (**B**), remain in remission whereas participants PaCa7 (**C**) and PaCa10 (**D**) relapsed. Adjuvant therapy is shown above the graph. Horizontal dotted line: CA19-9 critical limit (37 U/mL). GEM: gemcitabine; FOLFIRINOX: combination of 5-fluorouracil, irinotecan, and oxaliplatin

Longitudinal peripheral venous blood samples were collected and analyzed for CTCs in 4 participants with PDAC: PaCa6, PaCa7, PaCa10, and PaCa13 (Fig. 4). Two participants (PaCa6 and PaCa13) are currently disease-free at latest follow-up and had no CTCs detected in any post-surgery peripheral venous blood samples. Two participants (PaCa7 and PaCa10) who relapsed at 35 weeks and 57 weeks post-surgery respectively, had CTCs detected prior to clinical relapse and present in all of their post-surgery peripheral venous blood samples.

### CTC detection using Accucyte vs. CellSieve

**Fig. 5:**
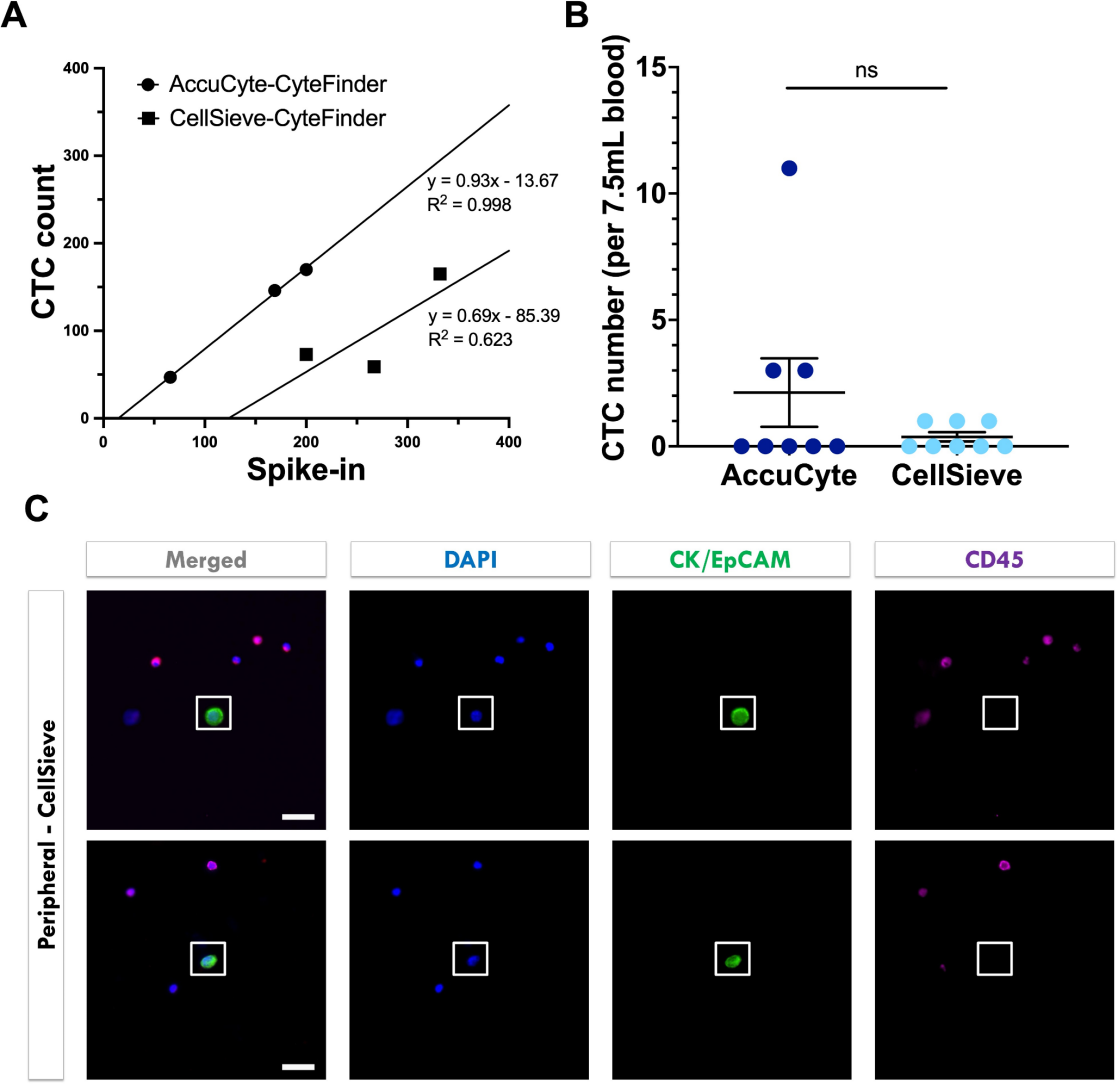
Circulating tumor cell (CTC) detection using AccuCyte or CellSieve. A: AccuCyte-CyteFinder had higher detection rates compared to CellSieve-CyteFinder in spike-in experiments. B: No significant (ns) difference in means for CTCs detected by AccuCyte or CellSieve in participants with pancreatic ductal adenocarcinoma. C: Representative images of CTCs identified by CellSieve. Magnification 40x, scale bar = 50μm.

CTC detection by AccuCyte and CellSieve microfiltration assays were compared with CyteFinder used in both methods for analysis. In surrogate CTC samples, AccuCyte exhibited significantly higher detection rates compared to CellSeive (81% vs. 36%, *p* = 0.009; linear regression slope: 0.69 vs. 0.93; Fig. 5A; Supp Table 4). An additional baseline peripheral venous blood sample was obtained from 8 patients in the epithelial cancer group and processed on CellSieve. The detection rate was 37.5% (3/8) for both AccuCyte and CellSieve (Supp Table 2) with no significant difference in means (2.125 vs. 0.375 respectively, *p* = 0.2; Fig. 5B-C).

## Discussion

This prospective observational pilot study examined baseline and longitudinal CTCs in those undergoing a Whipple procedure or partial pancreatectomy. Those with epithelial cancers had significantly higher detection rates of CTCs in baseline portal venous blood compared to peripheral venous blood samples (75% vs. 40%). CTCs were detected prior to clinical relapse in follow-up peripheral venous blood samples, and higher detection rates were found using the AccuCyte platform compared to the CellSieve platform in spiked-in samples but not clinical samples. Baseline portal venous CTC counts were more than 2.5 times higher than peripheral venous CTC counts suggesting that the portal vein being the direct drainage from the pancreas allows for greater number of CTCs to be detected. The presence of portal venous CTCs intraoperatively was predictive of recurrence with a relative risk of 6.67. Participants who relapsed post-surgery, had consistently detectable CTCs in their peripheral venous blood samples, even during adjuvant treatment, in a small cohort of participants. This signifies that CTCs from portal venous blood detected using the AccuCyte-CyteFinder platform may provide a potentially valuable biomarker for identifying pancreatic cancer patients at risk of relapse.

This is the first study, to our knowledge, to use the AccuCyte-CyteFinder platform to detect CTCs from portal venous blood samples. In those with resectable PDAC, portal venous blood resulted in a higher rate of detection of CTCs compared to peripheral venous blood (67% (10/15) vs. 27% (4/15) respectively) with no significant difference in the expression of CK or EpCAM on the detected CTCs. This supports previous studies in which higher detection rates and counts of CTCs in portal venous compared to peripheral venous blood samples were found [29–31, 33]. Although CTC detection in portal venous blood was lower compared to other studies (67% compared to 71–100% using CellSearch [27, 34], ClearCell [35] or IsoFlux [30]), this study demonstrated significant predictive risk stratification for early relapse with potential clinical application.

An unique aspect of our study is the inclusion of non-epithelial cancer and other malignancy samples, which act as controls for epithelial CTC detection. No CTCs were detected in those with non-epithelial cancer (pNET) or no malignancy with the exception of one participant with IPMN. This is expected, as CTCs were detected using epithelial markers only, and is important as it demonstrated good specificity (89% for portal venous; 100% for peripheral venous). For the participant with IPMN where CTC was detected, it is possible that this represent a potential malignant transformation of the precursor lesion, which can occur in up to 60% of main duct IPMNs [36]. However, further downstream analysis would be required to confirm the origin of the detected epithelial CTCs where the CyteFinder platform has an on-board cell picker for single cell mutation or proteomic analysis [37, 38], however this was out of the scope of the current study. Beyond the use of epithelial markers for detecting CTCs, cancer-specific markers and/or mesenchymal markers such as plectin [39], vimentin [40], EGFR [41] could be used or added to the existing panel to increase detection rates or increase prognostic utility.

The AccuCyte-CyteFinder platform was superior to the CellSieve microfluidic assay in spiked-in experiments. However, this was not replicated in clinical samples possibly due to the low CTC yield resulting in high sample-to-sample variability [42]. A limitation of portal venous sampling is that collecting large volumes intraoperatively can be challenging without increasing the risk of bleeding. As such, we were unable to evaluate CellSieve on portal venous blood samples. Our study demonstrated the use of the CyteFinder platform to analyse the CellSieve microfiltration filters, standardizing the analysis of the two different CTC detection methods. Our CTC detection rate of 37.5% for CellSieve is similar to those observed in other studies using the CellSieve microfiltration assay in pancreatic cancer (23–42%) [32, 43]. CTC clusters have been detected at a higher rate in breast cancer using CellSieve compared to CellSearch [24], however no CTC clusters were observed in our patient cohort using either CellSieve or the AccuCyte-CyteFinder platform.

This study identified portal venous CTCs to be predictive of relapse whilst none of the other clinicopathological factors were prognostic. It supports the investigation of portal venous CTCs using endoscopic ultrasound (EUS)-guided sampling, where several studies have found it to be safe and feasible [27, 28, 34]. However, this procedure is invasive and its role as a monitoring tool is still to be examined. Our study found that longitudinal monitoring of peripheral venous CTCs post-surgery was indicative of relapse, albeit in a very small cohort of patients. This requires validation in a larger cohort but suggests a role for CTCs as a monitoring tool for minimally residual disease, which has been shown in other cancers such as lung, breast and liver cancer [44–46] but not in PDAC so far.

Our findings hold significant clinical implications for the management of PDAC but also more broadly for patients undergoing Whipple or pancreatectomy procedures. The higher detection rates of CTCs in portal venous blood suggests that portal vein sampling could provide a more accurate assessment of tumor burden than peripheral venous blood. More importantly, our study identified CTCs in portal venous blood as a predictor of relapse, which has potential applications in screening patients for surgery and also tailoring post-operative management. Clinicians could identify patients at high risk of early relapse and consider more intensive adjuvant therapy, and by monitoring CTCs post-surgery, allow for early and timely intervention. CTCs could enable molecular profiling in near-real time to inform clinicians of the most effective treatment regimens, as demonstrated in ALK-mutant non-small cell lung cancer [47]. This represents a potentially decisive step toward more personalized treatment strategies, which is urgently needed in PDAC.

In summary, our study demonstrated higher rates and counts of CTCs detected from portal venous blood compared to peripheral venous blood in PDAC. Portal venous CTC enumeration provided useful prognostic information. Our study also found the potential for longitudinal CTC monitoring of minimal residual disease in PDAC. Further molecular characterisation of CTCs, such as KRAS mutational analysis, representing the most common mutation in pancreatic cancer [48], is required to confirm the origin of the detected CTCs. Other mutational or proteomic analysis could provide clinically useful insights for identifying potential targeted treatments. Larger, multicentre studies with longer follow-up are needed to confirm the predictive value of portal venous CTCs for relapse in PDAC with multivariate analyses. Longitudinal studies are also required to explore whether post-surgical CTC levels can be used to guide post-operative management and/or adjuvant therapy decisions. The integration of CTC monitoring into routine clinical practice could signify a major step towards personalized/precision medicine, leading to better outcomes in those diagnosed with PDAC.

## Electronic supplementary material

Below is the link to the electronic supplementary material.

Supplementary Material 1

## Data Availability

The dataset/s supporting the conclusions of this article are included within the article and/or included in supplementary data.
