# Supplementary material for: Portal venous circulating tumor cells as a biomarker for relapse prediction in resected pancreatic cancer
Source: Cell Mol Life Sci. 2025 Apr 10;82(1):155. doi: 10.1007/s00018-025-05669-x (PMC11985722; doi:10.1007/s00018-025-05669-x)
Supplement: Supplementary file 1 — Supplementary Material 1 [file 18_2025_5669_MOESM1_ESM.docx]

Supplementary Figures and Tables

Supp Figure 1. Portal venous circulating tumor cells significantly correlated with tumor stage. Mean + SEM. ** p<0.01

Supp Figure 2. Kaplan-Meier Curves for recurrence-free survival for portal venous blood (A) and peripheral venous blood (B) based on CTC positivity as defined by greater or equal to 5 CTCs per 7.5mL blood.

Supp Table 1. Clinical data of all study participants

| Study ID | Gender | Age | Histological grade | TMN stage | T size | LVI | PNI | Vas Recon | R | CA19-9 | Neoadjuvant | Adjuvant | Relapse (0=no, 1=yes) | PFS (Days) |
| --- | --- | --- | --- | --- | --- | --- | --- | --- | --- | --- | --- | --- | --- | --- |
| PaCa1 | M | 83 | PDAC | T1cN1 | 14 | Y | Y | N | R1 | 9 | - | - | 0 | 148 |
| PaCa2 | F | 57 | PDAC mod differentiated | T1cN0 | 11 | N | N | N | R0 | 9 | - | FOLFIRINOX | 0 | 202 |
| PaCa3 | M | 72 | PDAC mod differentiated | T1N1 | 10 | Y | Y | N | R0 | 458 | - | Capecitabine | 0 | 231 |
| PaCa4 | M | 75 | PDAC mod/poorly differentiated | T2N1 | 40 | Y | Y | N | R1 | 3 | - | - | 1 | 40 |
| PaCa5 | F | 67 | PDAC mod differentiated | T2N0 | 28 | Y | Y | Y | R0 | 14 | FOLFIRINOX & SBRT | FOLFIRINOX | 1 | 140 |
| PaCa6 | M | 70 | PDAC well differentiated | T2N0 | 27 | Y | Y | N | R0 | 21 | - | Gemcitabine/ Capecitabine | 0 | 1406 |
| PaCa7 | M | 68 | PDAC mod differentiated | T2N1 | 32 | Y | Y | N | R0 | 24 | - | FOLFIRINOX | 1 | 246 |
| PaCa8 | M | 77 | PDAC well differentiated | T2N2 | 25 | Y | Y | Y | R0 | 131 | - | Gemcitabine | 1 | 353 |
| PaCa9 | F | 67 | PDAC mod differentiated | T2N1 | 30 | Y | Y | N | R1 | 202 | - | - | 1 | 69 |
| PaCa10 | M | 80 | PDAC mod/poorly differentiated | T2N1 | 21 | Y | Y | N | R1 | 574 | - | Gemcitabine/ Capecitabine | 0 | 254 |
| PaCa11 | F | 74 | PDAC mod/poorly differentiated | T2N1 | 35 | Y | Y | Y | R0 | 636 | - | - | 1 | 192 |
| PaCa12 | F | 42 | PDAC mod differentiated | T2N2 | 40 | N | Y | Y | R0 | 779 | mFOLFIRINOX | FOLFIRINOX | 0 | 289 |
| PaCa13 | M | 74 | PDAC poorly differentiated | T2N1 | 24 | Y | Y | Y | R0 | 1810 | - | FOLFIRINOX | 1 | 339 |
| PaCa14 | M | 59 | PDAC | T3N2 | NA | NA | NA | NA | NA | 96 | - | Gemcitabine/ abraxane | - | NA |
| PaCa15 | F | 78 | PDAC poorly differentiated | T3N2 | 42 | Y | Y | Y | R0 | 498 | - | - | 1 | 26 |
| PaCa16 | M | 73 | Ampullary adenocarcinoma mod differentiated | T1N0 | 15 | N | N | N | R0 | 8 | - | - | 0 | 236 |
| PaCa17 | M | 66 | Ampullary adenocarcinoma mod differentiated | T1bN1 | 15 | Y | N | N | R0 | 11 | - | Capecitabine | 0 | 195 |
| PaCa18 | M | 66 | Intra-ampullary adenocarcinoma | T3N1 | 16 | Y | Y | N | R0 | 23 | - | Gemcitabine/ Capecitabine | 0 | 325 |
| PaCa19 | M | 66 | Bile duct adenosquamous carcinoma | T3N0 | 20 | Y | Y | N | R0 | 58 | - | - | 0 | NA |
| PaCa20 | M | 85 | Bile duct adenocarcinoma mod differentiated | T3N1 | 46 | Y | Y | N | R0 | 226 | - | - | 0 | 223 |
| PaCa21 | M | 80 | IPMN low grade | NA | NA | N | N | N | R0 | 5 | - | - | 0 | 200 |
| PaCa22 | M | 70 | IPMN | NA | NA | N | N | N | R0 | 12 | - | - | 0 | 1140 |
| PaCa23 | F | 55 | IPMN low grade | NA | NA | N | N | N | R0 | 20 | - | - | 0 | 174 |
| PaCa24 | F | 70 | PNET Grade 1 - mod differentiated | T1N0 | 16 | N | N | N | R0 | 9 | - | - | 0 | 284 |
| PaCa25 | M | 48 | PNET Grade 1 - well differentiated | T2N0 | 23 | N | N | N | R0 | - | - | - | 0 | 405 |
| PaCa26 | M | 78 | PNET Grade 2 | T2N1 | 32 | N | N | N | R0 | 13 | - | - | 0 | 343 |
| PaCa27 | F | 70 | GIST | NA | NA | NA | NA | N | R0 | 8 | - | - | 0 | 324 |
| PaCa28 | F | 32 | pseudopapillary neoplasm | T2N0 | 33 | N | N | N | R0 | 9 | - | - | 0 | 1139 |
| PaCa29 | M | 49 | serous cystadenoma | NA | NA | N | N | N | R0 | - | - | - | 0 | 111 |

Supp Table 2. CTC enumeration of study participants

|  | Peripheral Venous | | | |  | Portal Venous | | | |
| --- | --- | --- | --- | --- | --- | --- | --- | --- | --- |
| Study ID | Blood (mL) | Slides | CTC (slides) | CTCs in 7.5 mL |  | Blood (mL) | Slides | CTC (slides) | CTCs in 7.5 mL |
| PaCa1 | 7.5 | 8 | 0 (2) | 0 |  | 6.5 | 7 | 0 (2) | 0 |
| PaCa2 | 7.5 | 8 | 0 (2) | 0 |  | 7.5 | 8 | 1 (2) | 4 |
| PaCa3 | 7.5 | 8 | 0 (3) | 0 |  | 7.5 | 8 | 0 (2) | 0 |
| PaCa4 | 7.5 | 8 | 0 (3) | 0 |  | 7.5 | 8 | 1 (3) | 3 |
| PaCa5 | 7.5 | 8 | 0 (3) | 0 |  | 7.5 | 8 | 0 (3) | 0 |
| PaCa6 | 7.5 | 8 | 0 (3) | 0 |  | 6 | 8 | 1 (3) | 3 |
| PaCa7 | 7.5 | 8 | 4 (3) | 11 |  | 7 | 8 | 4 (3) | 11 |
| PaCa8 | 7.5 | 8 | 0 (3) | 0 |  | 7.5 | 8 | 3 (3) | 8 |
| PaCa9 | 7.5 | 8 | 0 (2) | 0 |  | 7.5 | 8 | 0 (3) | 0 |
| PaCa10 | 7.5 | 8 | 0 (3) | 0 |  | 7.5 | 8 | 0 (3) | 0 |
| PaCa11 | 7.5 | 8 | 1 (3) | 3 |  | 4 | 7 | 1 (2) | 7 |
| PaCa12 | 7.5 | 8 | 0 (3) | 0 |  | 6.5 | 7 | 2 (3) | 5 |
| PaCa13 | 7.5 | 8 | 6 (3) | 16 |  | 7.5 | 8 | 6 (3) | 16 |
| PaCa14 | 7.5 | 8 | 0 (3) | 0 |  | 7.5 | 8 | 5 (2) | 20 |
| PaCa15 | 7.5 | 8 | 5 (3) | 13 |  | 3 | 4 | 3 (2) | 15 |
| PaCa16 | 7.5 | 8 | 1 (3) | 3 |  | 7.5 | 8 | 1 (2) | 4 |
| PaCa17 | 5.7 | 6 | 0 (2) | 0 |  | 6.7 | 7 | 3 (2) | 12 |
| PaCa18 | 7.5 | 8 | 1 (3) | 3 |  | 7.5 | 8 | 12 (3) | 32 |
| PaCa19 | 7.5 | 8 | 6 (3) | 16 |  | 7.5 | 8 | 10 (3) | 27 |
| PaCa20 | 7.5 | 8 | 1 (3) | 3 |  | 4.2 | 5 | 2 (2) | 9 |
| PaCa21 | 7.5 | 8 | 0 (2) | 0 |  | 4.7 | 6 | 1 (2) | 5 |
| PaCa22 | 7.5 | 8 | 0 (3) | 0 |  | 7.5 | 8 | 0 (2) | 0 |
| PaCa23 | 7.5 | 8 | 0 (2) | 0 |  | 5.5 | 6 | 0 (2) | 0 |
| PaCa24 | 7.5 | 8 | 0 (3) | 0 |  | 7.5 | 8 | 0 (3) | 0 |
| PaCa25 | 7.5 | 8 | 0 (3) | 0 |  | 7.5 | 8 | 0 (2) | 0 |
| PaCa26 | 7.5 | 8 | 0 (3) | 0 |  | 5.5 | 6 | 0 (2) | 0 |
| PaCa27 | 7.5 | 8 | 0 (3) | 0 |  | 7.5 | 8 | 0 (3) | 0 |
| PaCa28 | 7.5 | 8 | 0 (3) | 0 |  | 7.5 | 8 | 0 (3) | 0 |
| PaCa29 | 7.5 | 8 | 0 (2) | 0 |  | 6 | 7 | 0 (2) | 0 |

Supp Table 3. Mean fluorescent intensity (MFI) value of identified circulating tumor cells from peripheral (Peri) and portal venous (PV) blood sample. Bolded are high expression (MFI >15)

| Study ID | Sampling | CK | EpCAM |
| --- | --- | --- | --- |
| PaCa2 | PV | **44.8** | 6.5 |
| PaCa4 | PV | **84.2** | 5.9 |
| PaCa6 | PV | 7.6 | **211.8** |
| PaCa7 | Peri | **165** | 7.1 |
|  |  | **99.2** | **10.9** |
|  |  | **97** | **12.2** |
| PaCa7 | PV | **87.2** | 7.7 |
|  |  | **164** | 6.2 |
|  |  | **108.2** | 8.1 |
|  |  | **71.9** | **10.9** |
|  |  | **70.1** | 6.7 |
| PaCa8 | PV | **84.2** | **19.3** |
|  |  | **283.9** | 7.2 |
|  |  | **37.1** | 4.8 |
|  |  | **27.7** | 5.1 |
| PaCa11 | Peri | **17** | **64.9** |
|  | PV | 4.5 | **60.7** |
| PaCa12 | PV | **622.3** | **890.6** |
|  |  | **840.7** | **16.5** |
| PaCa13 | Peri | **103.2** | 7.9 |
|  |  | **105.8** | 8.4 |
|  |  | 5.3 | **32.9** |
|  |  | **93.1** | **12** |
|  |  | **94.2** | **11.9** |
|  |  | **103.7** | 9 |
| PaCa13 | PV | **60.5** | 2.8 |
|  |  | **112.5** | **17.9** |
|  |  | **201.4** | 8.2 |
|  |  | **45** | 7.5 |
|  |  | **87.5** | **11.6** |
|  |  | **28.3** | 5.7 |
| PaCa14 | PV | **16.6** | **75.3** |
|  |  | **16.4** | **91.8** |
|  |  | **17.5** | **34.8** |
|  |  | **16.9** | **27.3** |
|  |  | 7 | **59.4** |
| PaCa15 | Peri | **82.5** | 8.5 |
|  |  | **24** | **10.1** |
|  |  | **35** | **14.7** |
|  |  | **11.8** | **99.5** |
|  |  | 8.4 | **103.1** |
| PaCa15 | PV | **40.5** | **2955.4** |
|  |  | **15.8** | **1897.7** |
|  |  | **23.9** | **2381.1** |
| PaCa16 | Peri | **28.8** | 8.7 |
| PaCa16 | PV | 5.9 | **62.1** |
| PaCa20 | Peri | **66.2** | 8.9 |
| PaCa20 | PV | **48.4** | 6.9 |
|  |  | **91.1** | 7.4 |

Supp Table 4. CTCs detected in intra-operative peripheral venous blood samples using AccuCyte and CellSieve

| Patient ID | Cancer | Stage | CTCs (in 7.5 mL blood) | |
| --- | --- | --- | --- | --- |
|  |  |  | AccuCyte | CellSieve |
| PaCa2 | PDAC | 1 | 0 | 0 |
| PaCa3 | PDAC | 1 | 0 | 0 |
| PaCa4 | PDAC | 2 | 0 | 1 |
| PaCa7 | PDAC | 2 | 11 | 0 |
| PaCa10 | PDAC | 2 | 0 | 1 |
| PaCa12 | PDAC | 2 | 0 | 1 |
| PaCa16 | AA | 1 | 3 | 0 |
| PaCa20 | BD | 3 | 3 | 0 |

Supp Table 3. Spike-in sample recovery rate for AccuCyte and CellSieve

| Healthy ID | Method | Spike-in | CTC count | Recovery (%) |
| --- | --- | --- | --- | --- |
| 9628 | AccuCyte | 169 | 146 | 86.3905325 |
|  | CellSieve | 332 | 164.5 | 49.5481928 |
| 9627 | AccuCyte | 200 | 170 | 85 |
|  | CellSieve | 200 | 73.25 | 36.625 |
| 9626 | AccuCyte | 66 | 47 | 71.2121212 |
|  | CellSieve | 267 | 59 | 22.0973783 |
|  |  |  |  |  |
